# Phenotype and Reactivity of Lymphocytes Expanded from Benign Prostate Hyperplasic Tissues and Prostate Cancer

**DOI:** 10.3390/cancers15123114

**Published:** 2023-06-08

**Authors:** Ritaparna Ahmed, Leyder Elena Lozano, Amandine Anastasio, Sebastien Lofek, Beatris Mastelic-Gavillet, Blanca Navarro Rodrigo, Sylvain Nguyen, Florence Dartiguenave, Sonia-Cristina Rodrigues-Dias, Valérie Cesson, Massimo Valério, Beat Roth, Lana Elias Kandalaft, Irina Redchenko, Adrian Vivian Sinton Hill, Alexandre Harari, Pedro Romero, Laurent Derré, Selena Viganó

**Affiliations:** 1 Department of Oncology, Centre Hospitalier Universitaire Vaudois, University Hospital of Lausanne, CH-1011 Lausanne, Switzerlandselena.vigano@debiopharm.com (S.V.); 2 Ludwig Institute for Cancer Research, University Hospital of Lausanne, CH-1011 Lausanne, Switzerland; 3 Urology Research Unit and Urology Biobank, Department of Urology, University Hospital of Lausanne, CH-1011 Lausanne, Switzerland; 4 Nuffield Department of Medicine, The Jenner Institute, Oxford University, Oxford OX3 7BN, UK

**Keywords:** benign prostatic hyperplasia, prostate cancer, vaccines, immunotherapy, tumor-specific T cells

## Abstract

**Simple Summary:**

Lymphocytes expanded from benign prostate hypertrophy (BPH) tissue samples recognize tumor antigens and autologous tissue independently from the presence of tumor lesions. These data sustain the connection between BPH and prostate cancer and may pave the way to personalized preventive vaccination in patients with BPH.

**Abstract:**

Benign prostate hyperplasia (BPH) is a frequent condition in aging men, which affects life quality, causing principally lower urinary tract symptoms. Epidemiologic studies suggest that BPH may raise the risk of developing prostate cancer (PCa), most likely promoting a chronic inflammatory environment. Studies aiming at elucidating the link and risk factors that connect BPH and PCa are urgently needed to develop prevention strategies. The BPH microenvironment, similar to the PCa one, increases immune infiltration of the prostate, but, in contrast to PCa, immunosuppression may not be established yet. In this study, we found that prostate-infiltrating lymphocytes (PILs) expanded from hyperplastic prostate tissue recognized tumor-associated antigens (TAA) and autologous tissue, regardless of the presence of tumor cells. PILs expanded from BPH samples of patients with PCa, however, seem to respond more strongly to autologous tissue. Phenotypic characterization of the infiltrating PILs revealed a trend towards better expanding CD4^+^ T cells in infiltrates derived from PCa, but no significant differences were found. These findings suggest that T cell tolerance is compromised in BPH-affected prostates, likely due to qualitative or quantitative alterations of the antigenic landscape. Our data support the hypothesis that BPH increases the risk of PCa and may pave the way for new personalized preventive vaccine strategies for these patients.

## 1. Introduction

Benign prostatic hyperplasia (BPH) is one of the most common diseases affecting aging men, causing lower urinary tract symptoms. The exact cause of BPH is not known, but it is thought to be related to changes in hormone levels as men age. Although controversial, BPH has been associated with prostate cancer (PCa) development [1]. Prostate cancer is the most common cancer in men, after skin cancer [2]. It is estimated that one in six men will develop prostate cancer in their lifetime in United States [2,3]. In patients affected by BPH, the gland is not only characterized by nonmalignant hyperplasia, but also by areas of chronic inflammation that share gene signatures and immune microenvironment with PCa [4]. Many cancers, including PCa, have been found to be strongly linked to prior inflammatory conditions [5,6]. In addition, morphological, epigenetic, and pharmacological evidences, together with large epidemiologic studies, indicate an increased risk of PCa in patients affected by BPH [1,7,8]. However, contradictory data highlight that (i) anti-inflammatory drugs have no effect on the risk of PCa development, (ii) BPH and PCa usually develop in different areas of the prostate, and (iii) BPH tissue has an age-driven low mutation rate [1,8]. Finally, predictive biomarkers of PCa development in patients with BPH are unknown. Thus, additional studies are needed to shed light on the connection between BPH and PCa.

The immune system plays a crucial role in the development and progression of BPH. Indeed, chronic inflammation is a hallmark of BPH, with increased infiltration of T cells, macrophages, and mast cells in the prostate tissue [9,10]. In line with chronic inflammation, BPH tissue exhibits a fundamental re-landscaping of immune infiltrates similar to PCa, showing a higher density of immune cells (mostly T cells) than healthy prostates [11,12]. The pro-inflammatory cytokines, such as tumor necrosis factor-alpha (TNFα), interleukin-6 (IL-6), and interleukin-8 (IL-8), are elevated in the serum and prostate tissue of BPH patients [13,14]. Understanding the interplay between the immune system and the prostate gland is essential for the development of new diagnostic and therapeutic approaches. Despite the lower abundance of somatic mutations and frequency of neoantigens in PCa than in melanoma or non-small cell lung cancer, tissue infiltrating lymphocytes (PILs) expanded from PCa recognize tumor-associated antigens (TAA) and autologous tissue [15,16]. Notably, TAA expression (e.g., PSCA) and the presence of genetic mutations (e.g., HBOX13) have been detected in BPH tissues at low levels and might be linked to an increased risk of developing PCa [8,17,18]. The immune response to BPH may be triggered by the release of antigens from dying prostate cells, leading to the activation of antigen-presenting cells and T cells. TAA and neoantigens expression in BPH tissues may result in the breakdown of T-cell tolerance. However, it is not known whether PILs derived from BPH tissues recognize TAA and autologous tissues. The goal of the present study is to determine the specificity of T cells recovered from BPH tissue, containing or not containing PCa. We found that PILs expanded from prostates, affected by BPH, recognize TAA and autologous tissue independently of the presence of PCa. Our data highlight a potential link between BPH and PCa in terms of T-cell specificity.

## 2. Materials and Methods

### 2.1. Samples

Freshly resected tissues were collected from patients with BPH undergoing transurethral resection of the prostate under the protocol 2019-00564, approved by the Ethics Committee of the Canton de Vaud (CER-VD; Switzerland). Informed consent from the patients was obtained based on the procedures approved by the CER-VD. This study was performed in compliance with the Declaration of Helsinki. Clinical characteristics are described in Table 1.

Freshly resected tissues not needed for histopathologic diagnosis were transferred in transport media (RPMI + 2% penicillin-streptomycin) in sterile containers at 4 °C. Prostate tissues were cut into 1–2 mm^2^ pieces and used freshly and/or as cryopreserved in 90% fetal bovine serum+ 10% DMSO, when possible.

### 2.2. Antibodies and Reagents

Zombie UV™ Fixable Viability Kit, anti-CD45 BV570, anti-CD3 BV605, anti-CD4 BV421, anti-CD8 BV650, anti-CD4 APC-Fire750, anti-CD73 PE/Dazzle 594, anti-CD56 PE, anti-CD14 PerCP/Cy5.5, anti-CD16 Alex Fluor 700, anti-CD19 APC-Fire750, anti-HLA-DR PE-Cy5, anti-PD-1 BV421, anti-CD16 PE, anti-IFNγ A700, anti-IL-13 APC, anti-IL-5 APC, anti-TNFα Alexa Fluor 488, and anti-IL-21 PE were purchased from BioLegend (London, UK). Anti-CD25 BB515, anti-CD4 BUV496, anti-CD39 BV711, anti-Perforin Alexa Fluor 647, anti-Granzyme B Alexa Fluor 700, anti-Ki-67 Alexa Fluor 700, anti-CD45RA PE, anti-IL-2 PerCP/Cy5.5, and Fixation/Permeabilization Solution Kit were purchased from Becton Dickinson. Anti-FOXP3 APC, anti-CD56 APC-eFluor780, and eBioscience™ (San Diego, CA, USA) Foxp3/Transcription Factor Staining Buffer Set were purchased from eBioscience TermoFisher scientific. Anti-TIM-3 APC was purchased from R&D systems. Anti-CD137 PE and FcR blocking reagent were purchased from Miltenyi (Bergisch Gladbach, Germany).

Peptides for the PSA, PSMA, PSCA, and PAP pools (Supplementary Table S1) were synthetized and lyophilized by the Peptide and Tetramer Core Facility of the Department of Oncology at UNIL-CHUV (Lausanne, Switzerland), and 5T4 pools (Supplementary Tables S2 and S3) were synthetized and lyophilized by JPT Peptide Technologies.

### 2.3. Expansion of Prostate Infiltrating Lymphocytes

Tissues were dissected into fragments of approximately 2 mm^3^. Each fragment was plated individually in a single well of a 24-well plate and stimulated with 6000 IU/mL rhIL-2 (Roche, Basel, Switzerland) for 3 weeks (Pre-REP). A rapid expansion protocol (REP) [15,19] was performed by stimulating PILs with PHA 1 µg/mL (Sigma, St. Loius, MI, USA), 3000 IU/mL rhIL2, and feeders. PILs culture media was RPMI supplemented with 5% penicillin-streptomycin (Gibco, Billings, MT, USA), 25 mM HEPES, 1% L-glutamine (Gibco), 1% nonessential amino acids (Gibco), 1% Na pyruvate (Gibco), 0.1% 2β-mercaptoethanol (Gibco), and 8% heat-inactivated human serum.

### 2.4. Cytokine Production Assay and Intracellular Staining

PILs were stimulated with anti-CD3/CD28 beads (bead-to-cell ratio = 1:2; Miltenyi) or tumor-associated antigen (TAA)-specific peptide pools (1 µM; Supplementary Tables S1–S3). Each stimulation was performed with only 1 pool [20]. Stimulation was performed overnight at 37 °C in the presence of GolgiPlug (1 µg/mL; Becton Dickinson, Franklin, NJ, USA). After washing, cells were stained for the surface markers CD3, CD4, and CD8 and with a viability dye for 20 min at 4 °C. Cells were then washed and permeabilized (30 min 4 °C, Fix and Perm buffer), and then they were washed and stained with antibodies directed to intracellular proteins (20 min, 4 °C). After washing, the cells were resuspended in PBS and analyzed by flow cytometry (LSRFortessa, BD Biosciences, Franklin, NJ, USA). Flow cytometry analysis was performed with FlowJo software (Version 10.2, Treestar, Woodburn, OR, USA).

### 2.5. PILs and Autologous/Heterologous Tissue Co-Cultures

An amount of 200,000 PILs/well were dispensed into a 96-well plate containing, or not containing, the same number of autologous or heterologous tissue cells recovered from cryopreserved prostate tissue. Cells were co-cultured overnight, then CD137 expression was measured by flow cytometry in CD4^+^ and CD8^+^ PILs [21].

### 2.6. Statistical Analysis

Statistical analysis was performed with Prism software (Version 7, GraphPad). We used nonparametric paired (Wilcoxon) tests. For multiple comparisons, adjusted *p*-values were calculated by ANOVA, followed by Dunn’s test or two-way ANOVA, followed by Dunnet’s test. Correlations were assessed by the nonparametric Spearman’s test.

## 3. Results

### 3.1. Phenotypic Characterization of Prostate Infiltrating Lymphocytes from BPH Tissue Samples

**Figure 1:**
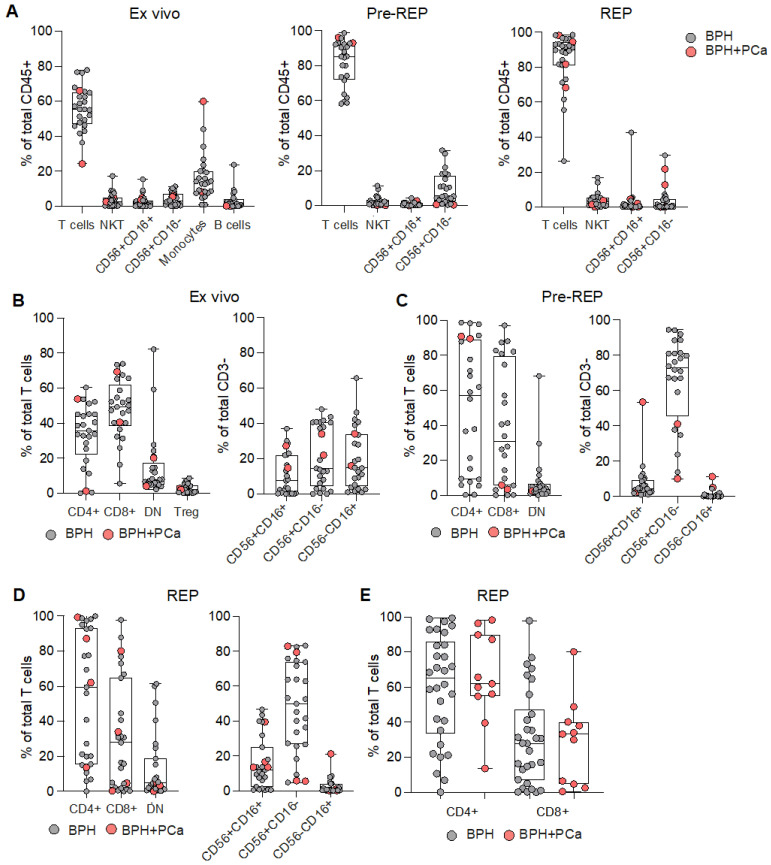
Characterization of lymphocytes infiltrating prostate tissues. (**A**) Frequency of the depicted populations among the total CD45^+^ cells measured ex vivo (left graph), after IL-2 mediated amplification (Pre-REP; central graph), or after rapid expansion protocol (REP; right graph). Analyzed tissues were affected by either benign prostate hyperplasia only (BPH; grey dots) or BPH with prostate cancer (BPH+PCa; pink dots). Proportion of CD4^+^, CD8^+^, CD4^neg^ CD8^neg^ double negative (DN) T cells, and CD4^+^ T regulatory cells (Treg; left graph), as well as subsets of NK cells (CD56^+^CD16^+^ CD56^+^CD16^−^ and CD56^−^CD16^+^; right graph) measured (**B**) ex vivo, (**C**) after pre-REP, or (**D**) after REP. (**E**) Proportion of CD4^+^ and CD8^+^ T cells composing total T-cells, which were expanded from BPH (grey dots) or BPH+PCa (pink dots).

We first collected prostate tissue from transurethral resection of the prostate (TURP) performed in patients with BPH (Table 1). Notably, during the analysis by the pathologist of prostate tissue specimen retrieved, besides BHP diagnosis, PCa was incidentally discovered (BPH+PCa) in some of them (Table 1). From these pieces of tissue, we generated prostate infiltrating lymphocytes (PILs) from TURP bulk specimen by in vitro culture with IL-2 (pre-REP) or using a rapid expansion protocol (REP) after pre-REP, as classically performed from tumor fragments [15,19]. We next characterized the phenotype of PILs directly ex vivo (after enzymatic digestion), after pre-REP, and after REP (Supplementary Figure S1a–c). Ex vivo immune cell infiltration was variable, reaching up to 20% of total cells (Supplementary Figure S1d), with the T-cell population being the most represented (Figure 1A). After expansion, T-cells accounted for more than 80% of total cells (Figure 1A), as expected. The CD4/CD8 ratio and the distribution of NK-cell subsets were variable among patient samples (Figure 1B,D). The composition of immune cells infiltrating tissues affected only by BPH or by BPH and PCa was not different (Figure 1A–D). We also evaluated the proportion of CD4^+^ and CD8^+^ T cells infiltrating prostate tissue after REP-expansion derived from additional 10 samples, containing only BPH and seven BPH samples, also containing PCa. T-cells expanded from BPH tissues, containing PCa foci, tended to have a higher proportion of CD4^+^ than CD8^+^ T cells, whereas PILs expanded from BPH samples had a more variable outcome (Figure 1E).

**Figure 2:**
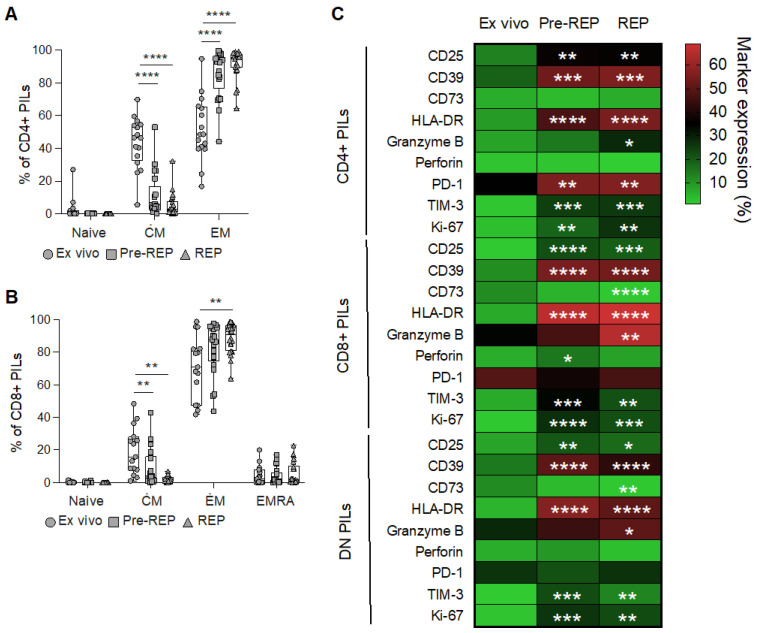
Phenotypic characterization of T cells infiltrating prostate tissues. Differentiation profile (CM: central memory cells; EM: effector memory cells: EMRA: effector memory cells re-expressing CD45RA) of (**A**) CD4^+^ and (**B**) CD8^+^ PILs measured ex vivo (dots), after IL-2 mediated amplification (Pre-REP; squares) or after rapid expansion protocol (REP; triangles). ANOVA tests, followed by Tukey’s tests. *P* values refer to differences from the ex vivo condition. ** *p* < 0.01, **** *p* < 0.0001. (**C**) Heatmap showing the frequency of expression of the depicted markers of activation/exhaustion and cytotoxicity by CD4^+^, CD8^+^, and DN prostate infiltration lymphocytes (PILs). ANOVA tests followed by Dunnett’s tests. *P* values refer to differences from the ex vivo condition. * *p* < 0.05, ** *p* < 0.01, *** *p* < 0.001, **** *p* < 0.0001.

Finally, consistent with previous findings [11], we observed a progressive differentiation of CD4^+^ and CD8^+^ T cells toward effector memory (EM) cells upon pre-REP and REP stimulation, at the expense of central memory cells (CM), while naïve and effector memory cells re-expressing CD45RA cells (EMRA) remain at a low frequency (Figure 2A,B and Supplementary Figure S1e–g). In addition, we measured the expression of a panel of activation/exhaustion and cytotoxic markers in T cells from prostate tissue ex vivo or after pre-REP and REP (Supplementary Figure S2). Almost all markers were increased after expansion (Figure 2C). Notably, we found a high expression of PD-1 in CD4^+^, as well as CD8^+^ T cells, from prostate tissue (Figure 2C), as shown previously [11].

### 3.2. Reactivity of Prostate Infiltrating Lymphocytes from BPH Tissue Samples

**Figure 3:**
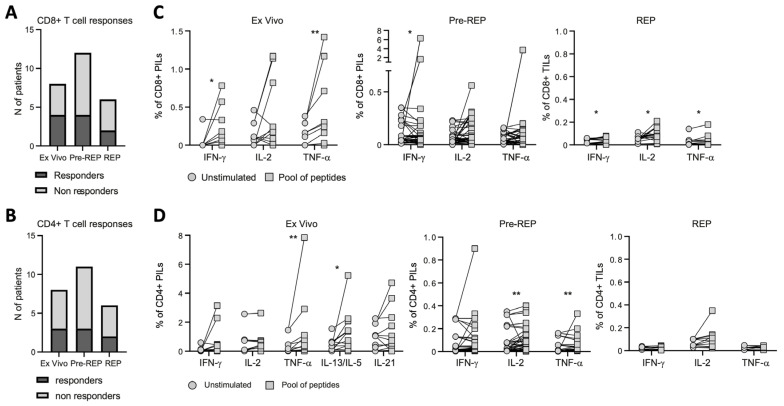
Prostate infiltrating T lymphocyte responses to tumor-associated antigen. Number of patients showing at least one CD8^+^ (**A**) or CD4^+^ (**B**) T-cell response to tumor-associated antigens (TAA). (**C**) Frequency of CD8^+^ prostate infiltrating lymphocytes (PILs) ex vivo (left graph), IL-2 amplified (Pre-REP; central graph), or after rapid expansion protocol (REP; right graph), producing IFNγ, IL-2, or TNFα, measured upon overnight stimulation with tumor-associated antigen (TAA) pools of peptides. (**D**) Frequency of CD4^+^ PILs ex vivo (left graph), IL-2 amplified (Pre-REP; central graph), or after rapid expansion protocol (REP; right graph), producing IFNγ, IL-2, TNFα, IL-13/IL-5, or IL-21 determined upon overnight stimulation by TAA pools of peptides. Indicated *p* values were determined by Wilcoxon tests. * *p* < 0.05, ** *p* < 0.01.

**Figure 4:**
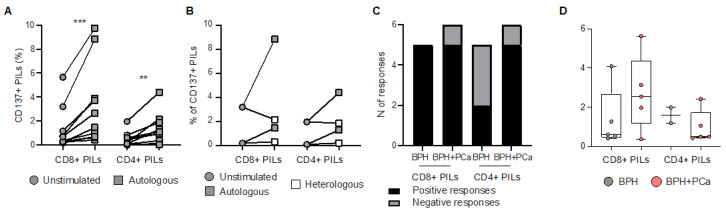
Prostate infiltrating T lymphocyte responses to prostate tissue. (**A**) Frequency of CD8^+^ and CD4^+^ prostate infiltrating lymphocytes (PILs), expanded by rapid expansion protocol (REP), expressing CD137 after overnight resting or stimulation by autologous enzymatically dissociated prostate tissue. (**B**) Frequency of CD8^+^ and CD4^+^ PILs, expanded by REP, expressing CD137 after overnight resting or stimulation by autologous or heterologous prostate tissue. (**C**) Number of positive (black) and negative (grey) responses, measured after overnight stimulation by autologous prostate tissue in CD8^+^ and CD4^+^ PILs, expanded from prostate samples affected by benign prostate hyperplasia (BPH) only or BPH and prostate cancer (BPH+PCa). (**D**) Increase (frequency after subtraction of background) in CD137 expression measured after overnight stimulation by autologous prostate tissue in CD8^+^ and CD4^+^ REP-expanded PILs derived from samples affected by BPH only (grey dots) or BPH+PCa (pink dots). Indicated *p* values were determined by Wilcoxon tests. ** *p* < 0.01, *** *p* < 0.001.

To investigate the functional properties and specificity of infiltrating T cells obtained from bulk BHP tissues, we assessed their ability to produce cytokines upon stimulation with pools of tumor associated antigens (TAA) derived peptides (i.e., PSA, PSMA, PSCA, PAP, and 5T4; Supplementary Tables S1–S3**)** or anti-CD3/CD28 antibodies. We analyzed PILs directly ex vivo, as well as after pre-REP and REP. Both CD8^+^ and CD4^+^ PILs responded to TAA-derived peptides ex vivo and after expansion, but the number and the magnitude of the responses were strongly reduced in REP cultures (Figure 3A–D and Supplementary Figure S3a), consistent with what we observed after polyclonal (anti-CD3/CD28 antibodies) stimulation (Supplementary Figure S3a,b). In order to understand whether PILs may recognize antigens presented by prostate tissue, PILs expanded from bulk BPH tissues were then cultured overnight with enzymatically dissociated prostate samples obtained from the same (autologous) or another (heterologous prostate tissue from BPH) patient. Stimulation with anti-CD3/CD28 was included as a positive control. Tissue-specific recognition was quantified as the frequency of CD137^+^ CD8^+^ PILs, measured by flow cytometry (Supplementary Figure S3c), given that CD137 is essential for identifying tumor-reactive lymphocytes [22]. Expanded PILs were activated when cultured with autologous tissue, but not when cultured with heterologous tissue (Figure S3a,b). These results suggest that tissue-specific T lymphocytes are present within BPH tissue. PILs were responding to autologous tissues independently of the presence of tumor cells (Figure 4C). However, the magnitude of the responses of CD8^+^ T cell infiltrating the prostate tissue obtained from patients incidentally diagnosed with PCa from BHP samples (BPH+PCa) tended to be greater than those measured in PILs expanded from samples affected only by BPH (Figure 4D), albeit we cannot confirm that those PILs were expanded from tissue fragments that actually contained PCa.

## 4. Discussion

Although different types of immune cells can be found, the majority of immune infiltrates from prostate tissues is composed of T cells, as previously shown [11]. As expected, the proportion of T cell becomes even higher upon expansion (pre-REP and REP), with an increase in the EM subset, at the expense of CM subpopulation [23]. Interestingly, a high expression of PD-1 was found ex vivo in T cells from BPH tissue. Since the expression of PD-1 by T cells from healthy prostate tissue and its overexpression in BHP and/or PCa tissue remains controversial [11,24,25], further studies are required to ascertain whether such high PD-1 expression we observed is a direct consequence of BPH or normal steady state. Nonetheless, it has been suggested that, instead of contributing to PCA-associated immune dampening, PD-1 may have a role in the regulation of prostate tissue homeostasis [11].

Our study also showed that T cells expanded from prostate tissue affected only by hyperplasia can recognize TAA and autologous tissues, similar to what has been observed in the presence of PCa in this study and by others [15,16]. These findings suggest that T-cell tolerance may be broken in BPH tissues, likely due to quantitative and/or qualitative alterations in expressed antigens, supporting the connection between BPH and PCa. Since PILs expanded from BPH tissue recognize autologous tissue, mutations generating immunogenic neoantigens might be present. The advancements in bioinformatics, as well as high-throughput sequencing technologies, have eased the discovery of neoantigens presented by tumor cells, rekindling interest in the development of personalized cancer vaccines. Investigation of the mutational and immunological landscapes of clearly histologically defined tissues (i.e., presenting BPH or BPH+PCa) will aid in elucidating the link between BPH and PCa diseases and, importantly, will provide novel immunotherapeutic opportunities for patients with BPH. Preventive immunization for BPH patients may be justified due to an increased risk of PCa development and the safe profile of vaccines. In addition, standard of care for patients with BPH is surgery, and few alternative treatment options are available for those patients, including alpha-blockers, 5α reductase inhibitors (5-ARIs), phosphodiesterase-5 (PDE5) inhibitors, and diuretics, further supporting immunization strategies [26,27]. Of note, preventive vaccines may also be more effective than therapeutic ones, since they do not face the immunosuppressive environment of established tumors. Personalized preventive vaccines may be of even higher interest in patients presenting small cancerous lesion in concomitance with BPH. Indeed, immunotherapy in prostate cancer has shown little efficacy, both when considering checkpoint inhibitors and vaccines [28,29,30]. Targeted and combinatorial therapies that aim at pulling multiple levers of the immune system may be the keys in providing successful treatment. Multiple clinical studies are ongoing to assess the potential therapeutic benefits of combining prostate cancer vaccines with immune-checkpoint blockades; early preliminary data show remarkable and long-lasting clinical response in some patients [31]. In patients affected by BPH with or without small PCa lesions, the immune system may be only partially immunosuppressed, and those combinations may even be more powerful. Indeed, our data show that autologous tissue recognition was present in PILs expanded from patients affected by BPH with or without concomitant PCa, and this was stronger in the second group. The capacity of those PILs to expand likely reflects the preservation of their functions. Overall, our findings suggest further research is warranted into whether BPH increases the risk of PCa.

## 5. Conclusions

Our data highlight that immune cells from BHP tissue are mainly composed of T cells, which highly expressed PD-1. Further studies are required to understand whether such high PD-1 expression is a direct consequence of BHP. In addition, our study reveals that T cells from BHP tissue recognize tumor antigens, as well as autologous tissues, independently from the presence of tumor lesions, suggesting a break in the T-cell tolerance in the BHP tissue.

## Figures and Tables

**Table 1:** Patients recruited in the study.

	BPH	BPH+PCa
Number of patients	44	12
Age, yr., median (IQR)	70 (66–77)	79 (70.75–82.75)
Concomitant chronic prostatitis	20	2
Gleason score	N/A	
6		4
7–8		3
≥9		5
Grade	N/A	
1–2		5
3–4		2
5		5

IQR = interquartile range. BPH = benign prostate hyperplasia. PCa = prostate cancer. N/A = not applicable.

## Data Availability

The datasets used and/or analyzed during the current study are available from the corresponding author on reasonable request.

## Supplementary Materials

The following supporting information can be downloaded at: https://www.mdpi.com/article/10.3390/cancers15123114/s1, Figure S1: Phenotypic characterization of lymphocytes infiltrating the prostate tissue; Figure S2: Expression of activation/exhaustion markers in prostate infiltrating lymphocytes; Figure S3: T cells infiltrating prostate tissue responses to TAA and prostate tissues; Table S1: Composition of pools for PSCA, PSA, PSMA and PAP proteins; Table S2: Composition of pools containing 10 amino acids peptides encompassing the 5T4 protein; Table S3: Composition of pools containing 15 amino acids peptides encompassing the 5T4 protein.
