# Supplementary material for: Phenotype and Reactivity of Lymphocytes Expanded from Benign Prostate Hyperplasic Tissues and Prostate Cancer
Source: Cancers (Basel). 2023 Jun 8;15(12):3114. doi: 10.3390/cancers15123114 (PMC10296351; doi:10.3390/cancers15123114)
Supplement: Supplementary file 1 [file cancers-15-03114-s001.zip › cancers-2403906-supplementary.pdf]

Supplementary Figure S1

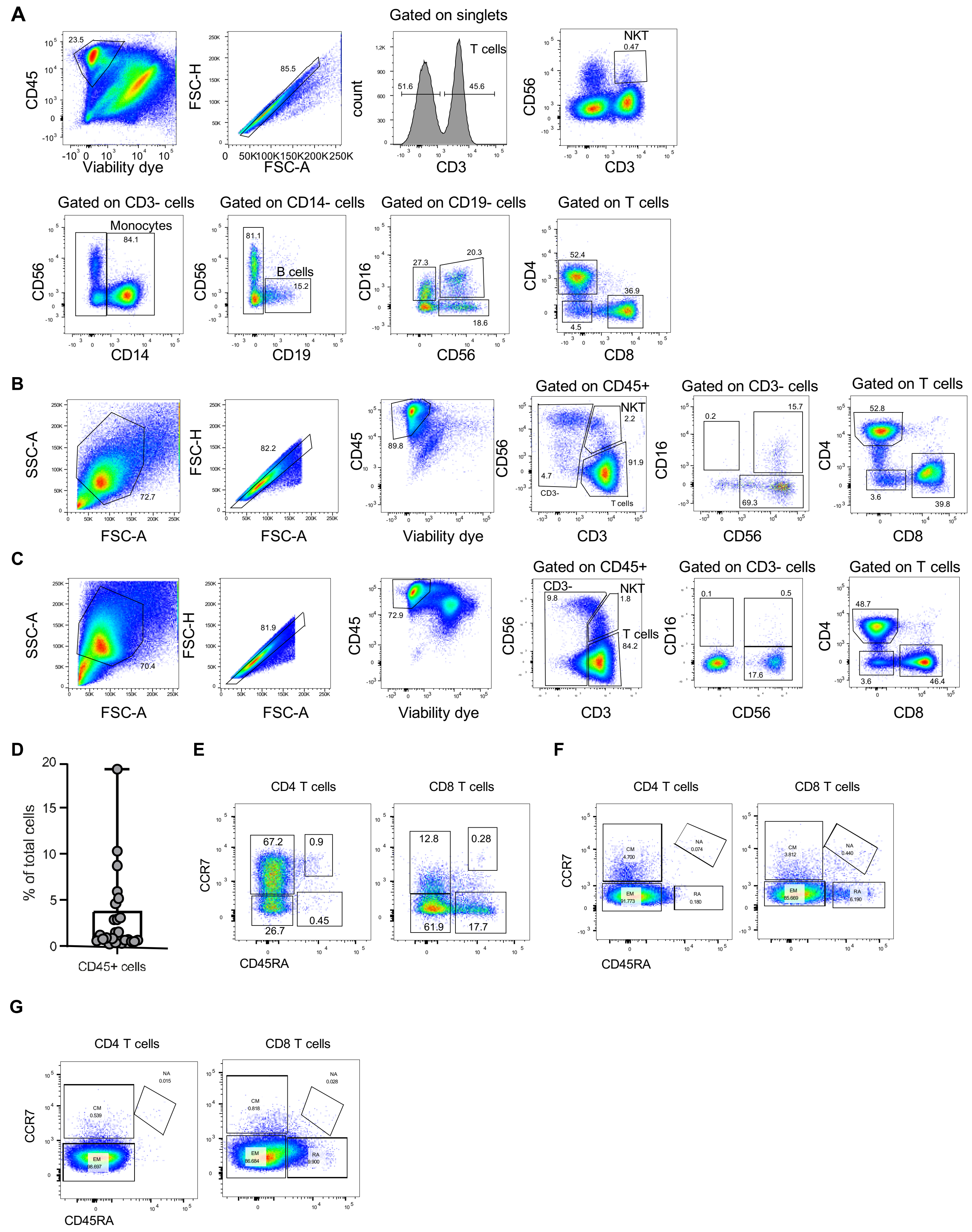

Supplementary Figure S1.Phenotypic characterization of lymphocytes infiltrating the prostate tissue.

Gating strategy for the quantification of the populations composing the prostate infiltrating lymphocytes (PILs) measured **(a)** *ex vivo*, **(b)** after IL-2 amplification (Pre-REP) and **(c)** after rapid expansion protocol (REP). **(d)** *Ex vivo f*requency of $CD45^{+}$ cells among the total live cells derived from prostate tissue digestion. Representative flow cytometry examples of the differentiation profile (CCR7 and CD45RA expression) of $CD4^{+}$ and $CD8^{+}$ T cells measured in PILs **(e)** *ex vivo*, **(f)** after Pre-REP, **(g)** after REP.

Supplementary Figure S2

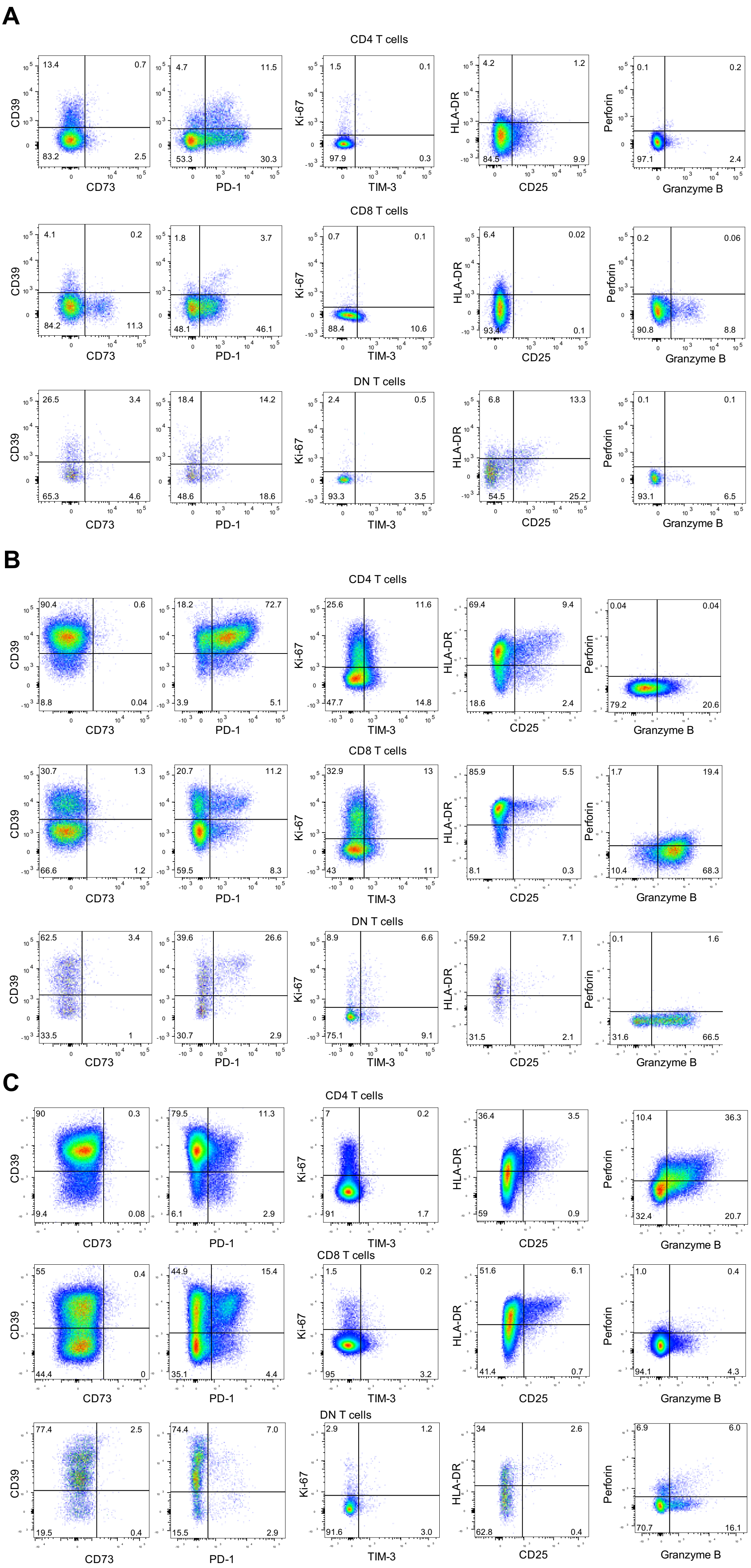

Supplementary Figure S2. Expression of activation/exhaustion markers in prostate infiltrating lymphocytes.

Representative flow cytometry examples of the expression of activation/exhaustion markers in $CD4^+$, $CD8^+$ and DN (double negative) T cells from prostate infiltrating lymphocytes (PILs) **(a)** *ex vivo*, **(b)** after IL-2 amplification (Pre-REP) and **(c)** after rapid expansion protocol (REP).

Supplementary Figure S3

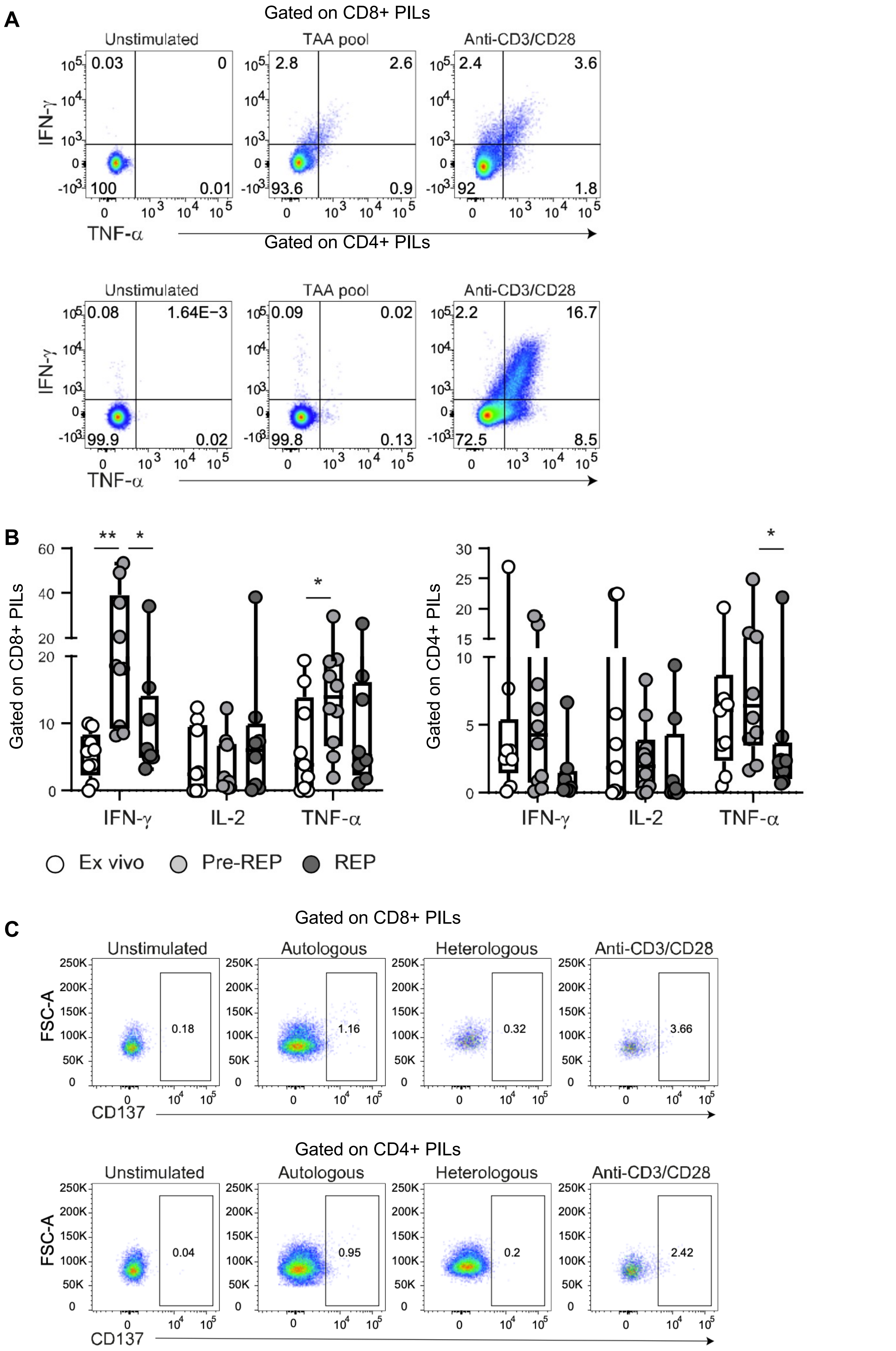

Supplementary Figure S3. T cells infiltrating prostate tissue responses to TAA and prostate tissues.
(a) Representative example of the response to tumor-associated antigens (TAA) or anti-CD3/CD28 stimulation by $CD8^+$ and $CD4^+$ IL-2 expanded (Pre-REP) prostate infiltrating lymphocytes (PILs). **(b)** Cumulative data of the anti-CD3/CD28 induced response in $CD8^+$ (left graph) and $CD4^+$ (right graph) PILs measured *ex vivo* (white dots), after Pre-REP (grey dots) and after REP (black dots). Kruskal-Wallis tests. *$p < 0.05$, **$p < 0.01$. **(c)** Representative example of the response to autologous or heterologous tissue or to anti-CD3/CD28 stimulation by $CD8^+$ and $CD4^+$ REP expanded PILs quantified by CD137 expression.

Supplementary Table S1. Composition of pools for PSCA, PSA, PSMA and PAP proteins

9-10 aminoacids peptides (HLA-A2)	
**Pool PSCA**	ALLMAGLAL
	ALQPGTALL
	LLCYSCKAQV
**Pool PSA**	VLVHPQWVL
	KLQCVDLHV
	FLTPKKLQCV
	VISNDVCAQV
**Pool PSMA**	LLHETDSAV
	VLAGGFFLL
	LLQERGVAYI
	MMNDQLMFL
**Pool PAP**	FLFLLFFWL
	TLMSAMTNL
	ALDVYNGLL

Supplementary Table S2. Composition of pools containing 10 aminoacids peptides encompassing the 5T4 protein

Sequences (206 peptides)						
MPGGCSRGPA	QCPALCECSE	AALNLSGSRL	NHIVPPEDER	LSNNSLVSLT	HMADMVTWLK	IVLALIGAIF
GGCSRGPAAG	PALCECSEAA	LNLSGSRLDE	IVPPEDERQN	NNSLVSLTYV	ADMVTWLKET	LALIGAIFLL
CSRGPAAGDG	LCECSEAART	LSGSRLDEVR	PPEDERQNRS	SLVSLTYVSF	MVTWLKETEV	LIGAIFLLVL
RGPAAGDGRL	ECSEAARTVK	GSRLDEVRAG	EDERQNRSFE	VSLTYVSFRN	TWLKETEVVQ	GAIFLLVLYL
PAAGDGRLRL	SEAARTVKCV	RLDEVRAGAF	ERQNRSFEGM	LTYVSFRNLT	LKETEVVQGK	IFLLVLYLNR
AGDGRLRLAR	AARTVKCVNR	DEVRAGAFEH	QNRSFEGMVV	YVSFRNLTHL	ETEVVQGKDR	LLVLYLNRKG
DGRLRLARLA	RTVKCVNRNL	VRAGAFEHLP	RSFEGMVVAA	SFRNLTHLES	EVVQGKDRLT	VLYLNRKGIK
RLRLARLALV	VKCVNRNLTE	AGAFEHLPSL	FEGMVVAALL	RNLTHLESLH	VQGKDRLTCA	YLNRKGIKKW
RLARLALVLL	CVNRNLTEVP	AFEHLPSLRQ	GMVVAALLAG	LTHLESLHLE	GKDRLTCAYP	NRKGIKKWMH
ARLALVLLGW	NRNLTEVPTD	EHLPSLRQLD	VVAALLAGRA	HLESLHLEDN	DRLTCAYPEK	KGIKKWMHNI
LALVLLGWVS	NLTEVPTDLP	LPSLRQLDLS	AALLAGRALQ	ESLHLEDNAL	LTCAYPEKMR	IKKWMHNIRD
LVLLGWVSSS	TEVPTDLPAY	SLRQLDLSHN	LLAGRALQGL	LHLEDNALKV	CAYPEKMRNR	KWMHNIRDAC
LLGWVSSSSP	VPTDLPAYVR	RQLDLSHNPL	AGRALQGLRR	LEDNALKVLH	YPEKMRNRVL	MHNIRDACRD
GWVSSSSPTS	TDLPAYVRNL	LDLSHNPLAD	RALQGLRRLE	DNALKVLHNG	EKMRNRVLLE	NIRDACRDHM
VSSSSPTSSA	LPAYVRNLFL	LSHNPLADLS	LQGLRRLELA	ALKVLHNGTL	MRNRVLLELN	RDACRDHMEG
SSSPTSSASS	AYVRNLFLTG	HNPLADLSPF	GLRRLELASN	KVLHNGTLAE	NRVLLELNSA	ACRDHMEGYH
SPTSSASSFS	VRNLFLTGNQ	PLADLSPFAF	RRLELASNHF	LHNGTLAELQ	VLLELNSADL	RDHMEGYHYR
TSSASSFSSS	NLFLTGNQLA	ADLSPFAFSG	LELASNHFLY	NGTLAELQGL	LELNSADLDC	HMEGYHYRYE
SASSFSSSAP	FLTGNQLAVL	LSPFAFSGSN	LASNHFLYLP	TLAELQGLPH	LNSADLDCDP	EGYHYRYEIN
SSFSSSAPFL	TGNQLAVLPA	PFAFSGSNAS	SNHFLYLPRD	AELQGLPHIR	SADLDCDPIL	YHYRYEINAD
FSSSAPFLAS	NQLAVLPAGA	AFSGSNASVS	HFLYLPRDVL	LQGLPHIRVF	DLDCDPILPP	YRYEINADPR
SSAPFLASAV	LAVLPAGAFA	SGSNASVSAP	LYLPRDVLAQ	GLPHIRVFLD	DCDPILPPSL	YEINADPRLT
APFLASAVSA	VLPAGAFARR	SNASVSAPSP	LPRDVLAQLP	PHIRVFLDNN	DPILPPSLQT	INADPRLTNL
FLASAVSAQP	PAGAFARRPP	ASVSAPSPLV	RDVLAQLPSL	IRVFLDNNPW	ILPPSLQTSY	ADPRLTNLSS
ASAVSAQPPL	GAFARRPPLA	VSAPSPLVEL	VLAQLPSLRH	VFLDNNPWVC	PPSLQTSYVF	PRLTNLSSNS
AVSAQPPLPD	FARRPPLAEL	APSPLVELIL	AQLPSLRHLD	LDNNPWVCDC	SLQTSYVFLG	LTNLSSNSDV
SAQPPLPDQC	RRPPLAELAA	SPLVELILNH	LPSLRHLDLS	NNPWVCDCHM	QTSYVFLGIV	
QPPLPDQCPA	PPLAELAALN	LVELILNHIV	SLRHLDLSNN	PWVCDCHMAD	SYVFLGIVLA	
PLPDQCPALC	LAELAALNLS	ELILNHIVPP	RHLDLSNNSL	VCDCHMADMV	VFLGIVLALI	
PDQCPALCEC	ELAALNLSGS	ILNHIVPPED	LDLSNNSLVS	DCHMADMVTW	LGIVLALIGA	

Supplementary Table S3. Composition of pools containing 15 aminoacids peptides encompassing the 5T4 protein

Sequences (82 peptides)	
MPGGCSRGPAAGDGR	GRALQGLRRLELASN
SRGPAAGDGRLRLAR	GLRRLELASNHFLYL
AGDGRLRLARLALVL	ELASNHFLYLPRDVL
LRLARLALVLLGWVS	HFLYLPRDVLAQLPS
LALVLLGWVSSSSPT	PRDVLAQLPSLRHLD
LGWVSSSSPTSSASS	AQLPSLRHLDLSNNS
SSSPTSSASSFSSSA	LRHLDLSNNSLVSLT
SSASSFSSSAPFLAS	LSNNSLVSLTYVSFR
FSSSAPFLASAVSAQ	LVSLTYVSFRNLTHL
PFLASAVSAQPPLPD	YVSFRNLTHLESLHL
AVSAQPPLPDQCPAL	NLTHLESLHLEDNAL
PPLPDQCPALCECSE	ESLHLEDNALKVLHN
QCPALCECSEAARTV	EDNALKVLHNGTLAE
CECSEAARTVKCVNR	KVLHNGTLAELQGLP
AARTVKCVNRNLTEV	GTLAELQGLPHIRVF
KCVNRNLTEVPTDLP	LQGLPHIRVFLDNNP
NLTEVPTDLPAYVRN	HIRVFLDNNPWVCDC
PTDLPAYVRNLFLTG	LDNNPWVCDCHMADM
AYVRNLFLTGNQLAV	WVCDCHMADMVTWLK
LFLTGNQLAVLPAGA	HMADMVTWLKETEVV
NQLAVLPAGAFARRP	VTWLKETEVVQGKDR
LPAGAFARRPPLAEL	ETEVVQGKDRLTCAY
FARRPPLAELAALNL	QGKDRLTCAYPEKMR
PLAELAALNLSGSRL	LTCAYPEKMRNRVLL
AALNLSGSRLDEVRA	PEKMRNRVLLELNSA
SGSRLDEVRAGAFEOH	NRVLLELNSADLDCD
DEVRAGAFEHLPSLR	ELNSADLDCDPILPP
GAFEHLPSLRQLDLS	DLDCDPILPPSLQTS
LPSLRQLDLSHNPLA	PILPPSLQTSYVFLG
QLDLSHNPLADLSPF	SLQTSYVFLGIVLAL
HNPLADLSPFAFSGS	YVFLGIVLALIGAIF
DLSPFAFSGSNASVS	IVLALIGAIFLLVLY
AFSGSNASVSAPSPL	IGAIFLLVLYLNRKG
NASVSAPSPLVELIL	LLVLYLNRKGIKKWM
APSPLVELILNHIVP	LNRKGIKKWMHNIRD
VELILNHIVPPEDER	IKKWMHNIRDACRDOH
NHIVPPEDERQNRSF	HNIRDACRDHMEGYOH
PEDERQNRSFEGMVV	ACRDHMEGYHYRYEI
QNRSFEGMVVAALLA	MEGYHYRYEINADPR
EGMVVAALLAGRALQ	YRYEINADPRLTNLS
AALLAGRALQGLRRL	NADPRLTNLSSNSDV
